# Impacts of limits to adaptation on population and community persistence in a changing environment

**DOI:** 10.1098/rstb.2023.0322

**Published:** 2025-01-09

**Authors:** Luis-Miguel Chevin, Jon Bridle

**Affiliations:** ^1^ CEFE, University of Montpellier, CNRS, EPHE, IRD, Montpellier, France; ^2^ Department of Genetics, Evolution and Environment, University College London, London, UK

**Keywords:** evolutionary rescue, ecological margins, genetic architecture, phenotypic plasticity, fluctuating environment, fitness function

## Abstract

A key issue in predicting how ecosystems will respond to environmental change is understanding why populations and communities are able to live and reproduce in some parts of ecological and geographical space, but not in others. The limits to adaptation that cause ecological niches to vary in position and width across taxa and environmental contexts determine how communities and ecosystems emerge from selection on phenotypes and genomes. Ecological trade-offs mean that phenotypes can only be optimal in some environments unless these trade-offs can be reshaped through evolution. However, the amount and rate of evolution are limited by genetic architectures, developmental systems (including phenotypic plasticity) and the legacies of recent evolutionary history. Here, we summarize adaptive limits and their ecological consequences in time (evolutionary rescue) and space (species’ range limits), relating theoretical predictions to empirical tests. We then highlight key avenues for future research in this area, from better connections between evolution and demography to analysing the genomic architecture of adaptation, the dynamics of plasticity and interactions between the biotic and abiotic environment. Progress on these questions will help us understand when and where evolution and phenotypic plasticity will allow species and communities to persist in the face of rapid environmental change.

This article is part of the discussion meeting issue ‘Bending the curve towards nature recovery: building on Georgina Mace's legacy for a biodiverse future’.

## 1. Introduction

Current rates of environmental change pose risks to population persistence, the composition of ecological communities, and the stability and reliability of the outputs that ecosystems generate. A better understanding of the mechanisms through which environmental change affects population growth and stability can help better predict, and hopefully influence through intervention, the fates of populations and communities in response to changing environments. Because phenotypic traits mediate how organisms interact with their environments and perform within them [1,2], phenotypic change in populations under the combined contributions of genetic evolution and phenotypic plasticity, and their influences on demographic vital rates, should determine when and why populations persist under novel environmental conditions [3,4]. Conversely, understanding the limits that make local adaptation and adaptive plasticity insufficient to ensure population persistence in some conditions is key to predicting ecological margins and extinction risk in individual species, with concomitant effects on community stability and ecosystem functioning.

The idea that limits to adaptation are the fundamental cause of the finiteness of ecological niches and so determine the stability of ecological communities and the functioning of ecosystems under environmental change was something that Georgina Mace (1953–2020) returned to throughout her career. In her view, adaptive limits were both a key driver of broad macro-ecological scaling laws and a determinant of which life-history traits and taxonomies are most associated with high extinction risk. Reciprocally, this non-randomness of extinction with respect to traits and evolutionary (genomic) histories also makes it much more likely that biodiversity loss will cause the disappearance of whole branches rather than twigs in the tree of life, with the associated loss of key functions in ecological communities [5]. This meant that Georgina Mace persistently championed the importance of biodiversity within populations and adaptive divergence among populations across species’ geographical ranges (now highlighted as ‘cryptic biodiversity’ [6]), both for current ecosystem functioning and for evolution in future, novel environments [7]. After she became Director of the Centre for Population Biology (Imperial College London) in 2006, Georgina was instrumental in appointing the population and quantitative geneticist Russ Lande there. This led to a collaboration with him (and one of us) on the role of phenotypic plasticity and evolution in predicting population persistence in changing environments [3]. Georgina then included limits to adaptation to environmental change as one of the three research themes as the inaugural Director of the Centre for Biodiversity and Environment Research (CBER) following her move to University College London in 2013.

In line with these efforts to bridge macro-ecological observations with population-level biological mechanisms, there has been a flowering of theoretical and empirical work in evolutionary demography (currently often described as eco-evolutionary dynamics), aiming to predict the influence of a changing environment on species’ extinction risk, community functioning and ecosystem stability, through its effect on changes in allele and genotype frequencies. Here, we will mostly cover work on within-species dynamics, although we also address the influence of species’ interactions in the final section. We focus on the demographic and ecological circumstances in which alleles and genotypes can spread and be maintained to allow adaptation to novel environments (‘soft’ or ‘population genetic’ limits, *sensu* [8]) and leave discussion of larger scale genomic or phenotypic innovations that can break apart long-standing trade-offs in physiology or organismal design (‘hard limits’, *sensu* [8]) to another article in this issue [9].

Even when focusing on environments that change over time (e.g. due to climate change and other directional anthropogenic alterations), spatial considerations cannot be ignored, given that most species are distributed over geographic ranges with heterogeneous environments, leading to spatial variation in selection, demography and (often) genetic composition. When considered within such a spatial context, temporal environmental change not only modifies the most common environments experienced at the core of a species’ range but also (crucially) causes environments that were previously extreme (either rare throughout and/or only found at the margins of the distribution) to become increasingly common. For this reason, much can be understood about limits to adaptation in time by focusing on what happens towards the margins of existing species’ distributions (classic space-for-time substitution [10]). Range margins (especially those at lower latitudes) are where current climate change is likely to first expose a species to the newest environments relative to its evolutionary history, and therefore where the most rapid adaptive evolution will need to take place. Beyond the more extreme environments they experience, range margins also have specific demographic and genetic attributes, such as lower population size or density, higher levels of outbreeding (and sometimes also inbreeding depression), lower effective population size and local increases or decreases in gene flow [11,12], which need to be understood and accounted for when predicting limits to adaptation.

To summarize our understanding of adaptive limits and their role in determining species’ distributions and extinction risk in a changing environment, we first synthesize theoretical predictions, before reviewing empirical tests of this theory, paying particular attention to the challenges in translating models into actual biology, especially in natural populations. This leads us to identify key research needs to improve the prediction of population and community responses to changing environments.

## 2. Theory on limits to adaptation and population persistence

Theory in evolutionary ecology has long modelled adaptation as the match between phenotypes/genotypes and the environment, and connected evolutionary fitness to demographic vital rates [13–16]. However, evolutionary demographic models that directly ask how limits to the rate of adaptation determine extinction risk flourished especially during the 1990s [17–19], given the growing awareness of climate change at the time, followed by a surge of models 20 years later. The focus of this theory is not only on populations with immediate conservation concerns, where maladaptation would be the direct cause of extinction. Instead, its aim is to understand when and how environmentally induced maladaptation can drive initially sustainable populations towards much lower abundances, where they experience a higher risk of extinction. Extinction *per se* might then eventually occur for reasons not related to the changing environment (e.g. demographic stochasticity, Allee effects, inbreeding depression, etc. [20]). However, in the initial phase of population decline caused by environmental change, limits to adaptation are critical to the demographic outcome.

### (a) Theory on evolutionary rescue and sustainable rates of environmental change

**Figure 1.**
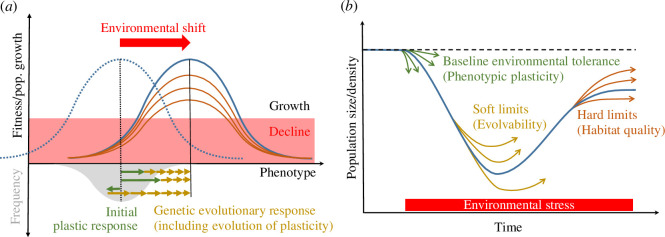
Adaptation limits and ER in a new environment. The main phenotypic (*a*) and demographic (*b*) consequences of an abrupt shift to a new stressful environment are illustrated. (*a*) Top: absolute fitness (population growth rate) depends on the phenotype through the fitness function (blue and orange curves). This causes some phenotypes (and their underlying genotypes) to lead to a demographic decline in any environment (red-shaded area). Environmental change shifts the fitness function from the dotted to the continuous blue curve, moving the optimum phenotype to the right (black vertical lines). The height of the fitness peak may also change (orange curves) if the new environment imposes hard limits that cannot be overcome by the arrangement of existing alleles, or those accessible by mutation. Bottom: the initial phenotypic distribution (grey-shaded area) is centred on the optimum in the original environment (dotted black line), but some individuals deviate from this optimum, leading to a standing load proportional to phenotypic variance around the optimum that reduces mean population growth, even if the population mean matches the optimum. After the environment changes, the mean phenotype is displaced from the new optimum (continuous black line), leading to a lag load (proportional to the deviation of the mean phenotype from the optimum), which here causes additional demographic cost and population decline (fitness enters the red-shaded area in top panel). The mean phenotype may undergo a rapid plastic phenotypic response in the first generation(s) after environmental change, represented by the green arrows, which may reduce this lag load (although note that the last arrow pointing to the left represents maladaptive plasticity, increasing lag load). Evolutionary response by cumulative changes in genotype frequencies under natural selection follows in subsequent generations (yellow arrows), eventually restoring positive population growth when ER is successful. (*b*) The population dynamics resulting from the processes depicted in (*a*) are illustrated by the blue line. Before the onset of environmental stress, the population is at equilibrium near the carrying capacity of the original environment, with population density reduced only by standing load due to phenotypic variance around the optimum. Environmental stress (illustrated by the red zone above the *x*-axis) causes an initial population decline, which may be attenuated by adaptive (or amplified by maladaptive) plasticity, determining the initial environmental tolerance of the species. The rate of evolutionary change later determines how fast a positive growth rate is restored (and what minimal size is reached), as illustrated by the yellow arrows, and is subject to soft limits to adaptation (i.e. factors influencing the pace of changes in allele and genotype frequencies). After the population has rebounded, it may reach a lower population size at equilibrium (orange arrows) because of hard limits in the novel environment that need to be overcome by rare (i.e. distant in space or time) alleles or mutations.

Models that do not explicitly account for space have investigated how different patterns of environmental change over time influence extinction risk in a single population and how this outcome may be prevented by adaptive evolution. Outputs from these models demonstrated from the outset that limits to adaptation are critical for population persistence in a changing environment. Gomulkiewicz & Holt [18] first coined the term Evolutionary Rescue (hereafter ER) to describe the situation where a population avoids extinction through evolution. They focused on abrupt environmental change (e.g. when physical systems such as climate cross tipping points, or following biological introductions), initially causing severe maladaptation and population decline (figure 1). By contrasting a classic quantitative genetic model of a heritable polygenic trait adapting to a shifted optimum phenotype (as in figure 1) with a simpler one-locus bi-allelic genetic architecture of adaptation, they showed that the probability of ER depended not only on the strength of selection (related to the intensity of stress), but also, crucially, on the amount of genetic variance, and how the latter is distributed among loci. Subsequent studies have explored these adaptive limits in more detail, by investigating the influence of diverse genetic architectures [21], selective interactions among loci (fitness epistasis) [22], genetic correlations among traits [23] and properties of the underlying developmental system (including the structure of gene-regulatory networks), broadly summarized as its evolvability [24]. A key conclusion from these studies is that ER is more likely where genetic systems enable higher rates of adaptive evolution, provided that this does not come at the expense of higher genetic load (due to mutation, recombination, etc.). In particular when considering adaptation to a shifted optimum phenotype, larger genetic variance causes faster adaptation, reducing the lag load due to the mean mismatch with the optimum, but may also increase the standing load due to phenotypic variance around that optimum (figure 1; reviewed in [25]).

**Figure 2.**
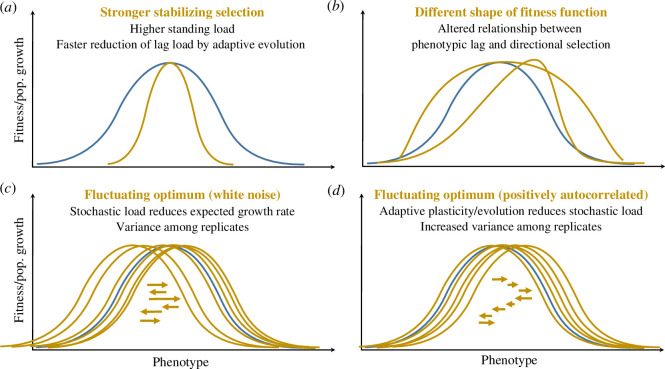
The shape of the fitness function and its effects on rates of adaptation. The baseline fitness function that relates a phenotypic trait to fitness is represented in blue, while yellow curves represent different modifications of this phenotype-to-fitness map. (*a*) A narrower fitness peak leads to a stronger stabilizing selection. This causes a higher standing load in a constant environment because even when the mean phenotype is at the optimum, individuals with sub-optimal phenotypes incur a higher fitness cost than those with a wider fitness peak. However, in a changing environment, directional selection on the mean phenotype is stronger with a narrower fitness peak, leading to faster evolution and a reduced lag load. (*b*) A different shape of the fitness function means that the relationship between the fitness cost of maladaptation (lag load) and the strength of directional selection is modified. Substantial maladaptation may therefore accumulate without leading to sufficient evolutionary response. (*c*) Fully unpredictable random fluctuations in the optimum cause an additional, stochastic component of lag load, reducing the expected long-term growth rate of the population, because the mean phenotype is unlikely to be at the optimum in any generation. Stochasticity also causes chance variation in eco-evolutionary outcomes (deviation of mean trait from the optimum, population growth rate, etc.), which has to be accounted for to predict, for example, extinction risk. (*d*) When random environmental fluctuations are autocorrelated in time, then they are partly predictable and can be tracked by adaptive plasticity. Positive autocorrelation also means that the direction of selection is likely to remain the same over multiple generations, making adaptive evolution more efficient in reducing mismatches with the optimum. Both effects cause positive autocorrelation to reduce the expected lag load generated by environmental fluctuations. However, positive autocorrelation also increases chance variation among eco-evolutionary trajectories (relative to uncorrelated stochastic fluctuations), because it leads to strings of bad or good environments, which also influences demography and extinction risk.

Models that focus on gradual directional changes in the environment (such as deterministic rises in mean global temperatures), where the key predictor is the maximum rate of environmental change that a population can sustain without going extinct [17,26,27], also emphasize the importance of limits to the rate of adaptation. The crucial role of genetic parameters such as rates of mutation [27] and recombination [28] has also been highlighted in this context. Furthermore, recent theory on this scenario has pointed out that the shape of the fitness function relating phenotypic traits to demographic vital rates can also be critical to population persistence by adaptation to the changing environment. In particular, fitness functions that break the one-to-one mapping between maladaptation (mean deviation from the optimum phenotype) and the strength of directional selection ‘pulling’ the mean phenotype towards the optimum (figure 2*b*) can result in evolutionary tipping points, beyond which maladaptation increases without bounds and leads to extinction [29,30].

Three threads of ongoing research emerge from recent work on ER in a gradually changing environment. The first involves modelling demography more explicitly. In particular, the role of demographic stochasticity (randomness in birth and death events) in the fate of initially rare novel mutations that may lead to ER has been emphasized, especially in the literature on microbes, whose very large population sizes make it very likely that *de novo* mutations make a significant and rapid contribution to ER [31–36]. This body of literature makes it clear that the range of stress levels over which ER from *de novo* mutations is possible (rather than persistence or extinction regardless of evolution) is relatively narrow [31,32]. This is even more true when the proportion of potentially rescuing mutations decreases with stress harshness (measure by, e.g. the decline rate of the wild-type), as expected under a fitness landscape with an optimum [33]. Another element of demographic realism that has been included in models of ER is density dependence in population growth (and possibly also in selection) [30,37,38], with contrasting interpretations about its impacts. On one hand, density dependence further reduces population growth (in addition to the detrimental effect of maladaptation), amplifying net population decline at a given distance from the optimum, thus diminishing the prospects for ER [37]. On the other hand, the decline of the wild-type population leads to reduced density-dependent competition, which can increase the growth rate of a rare rescue mutation and thus favour its emergence by escaping genetic drift (competition release hypothesis [38]).

A second thread of recent research on ER involves responses to randomly fluctuating environments (figure 2*c,d*). Although some of the earlier models of ER did include stochastic environmental fluctuations (environmental noise) in addition to deterministic trends [17,27,39], they did not explore in detail how different patterns of noise affect the evolutionary demographic outcome. In particular, global climate change impacts not only the magnitude but also the predictability of environmental noise [40,41]. The latter can be quantified by its temporal autocorrelation function, sometimes summarized as the ‘colour’ of environmental noise [42]. White noise characterizes completely unpredictable fluctuations, red noise describes positive autocorrelation—where subsequent timesteps have similar environments—and blue noise is for negative autocorrelation—where timesteps alternate from above to below the mean environment. Environmental autocorrelation can have opposing effects on the prospects for ER in a stochastic environment [43]. On the one hand, when a randomly fluctuating environment causes movements of an optimum phenotype, higher positive autocorrelation (more predictable environmental fluctuations) facilitates tracking of the moving optimum through adaptive evolution, so increasing expected mean fitness and population growth by reducing the so-called stochastic lag load [39,44]. On the other hand, higher positive autocorrelation of population mean fitness across time leads to higher variance among evolutionary demographic trajectories, increasing the probability that some replicates go extinct, even when ER would be expected deterministically [43] (figure 2*d*). Conversely, in situations where extinction would be very likely without stochasticity, autocorrelated environmental noise may increase the rescue probability, because it means that some stochastic paths will encounter by chance a series of less detrimental environments than the average [45].

The third thread of recent research on ER involves broadening the mechanisms underlying phenotypic variation and inheritance across environments. In particular, acknowledging that rapid trait change in natural populations typically includes a substantial contribution from phenotypic plasticity [46–48] (where a given genotype produces multiple phenotypes in response to the environment) has stimulated theory on the role of constant [3] or evolving plasticity [37,49,50] in ER. This theory (reviewed in detail elsewhere [51–53]) has formalized and quantified earlier verbal arguments about the important positive contribution of adaptive plasticity, and possibly its evolution, to population persistence and adaptation to novel environments [54–56]. In a randomly fluctuating environment, whether the demographic impact of plasticity is beneficial or detrimental depends on how well plasticity matches environmental predictability. Mismatched plasticity, where the reaction norm slope is higher or lower than required by the degree of environmental predictability, increases extinction risk by amplifying the stochastic lag load [57]. But if plasticity evolves fast enough towards matching environmental predictability, ER can occur by the rapid evolution of plasticity [49]. Note however that these theoretical predictions largely depend on a simple model of plasticity (i.e. a linear reaction norm for a fixed trait), as we elaborate in a later section.

### (b) Theory on adaptation across spatial environmental gradients and geographic range limits

Theory has also thoroughly explored the ecological consequences of adaptation limits when environments vary in space, rather than time. These are summarized by Bridle & Hoffmann [8], who argue that limits to local distributions typically result from ‘soft’ (population genetic) limits rather than ‘hard’ ones (novel mutations and traits), because they reflect when and where adaptive genotypes and phenotypes can actually persist within the range of a species’ maximum ecological tolerances, regardless of historical explanations based on barriers to dispersal or biogeography.

Haldane [58] first pointed out verbally that gene flow from the centre of a species’ range may prevent adaptation at its margins, where the selection pressures are likely to differ. This phenomenon prevents further expansion of the range, confining species to narrower sections of ecological space than could be occupied if genotypes were free to assemble and evolve in response to selection. Haldane’s verbal argument was turned into a mathematical model of the evolution of the species’ range in a classic study by Kirkpatrick & Barton [19] (based on the formalism of Pease *et al.* [26]), who modelled a set of populations spread over a spatial environmental gradient (such as the latitudinal or elevational gradients of temperature) that causes a cline in the optimum phenotype for a quantitative trait. They showed that gene flow causes more maladaptation in smaller populations at the margins, because they receive proportionally more migrants, reducing their mean fitness and population size. This further increases the asymmetry in gene flow and maladaptation, leading to a collapse of adaptation and population persistence at the margins, through a feedback between demography and evolution. In this first version of the model, adaptation was limited by the deterministic swamping effect of gene flow on mean phenotypes (similar to its effect on alleles at individual loci [59]). However, later studies that also allowed genetic variance to increase due to gene flow along a cline (reviewed in e.g. [60]) showed that this inflation of genetic variance proved more crucial to the eco-evolutionary feedback over space. This is because the increase in local genetic variance through gene flow among genetically differentiated populations leads to higher adaptive potential in each local population. This in turn allows adaptation to even very steep environmental gradients in deterministic models that neglect genetic drift, especially under polygenic architectures where the variance inflation is likely to be large [61–64]. On the other hand, the variance inflation generated by gene flow [61] also reduces mean fitness and population size, by causing individuals to deviate from the optimum even when the mean phenotype does not (standing load). This reduced effective population size results in increased genetic drift, hampering allele frequency change by natural selection, thus setting limits to local adaptation and leading to finite ranges (reviewed in [60]). This means that the positive feedback highlighted in earlier models [19], involving the swamping effect of (asymmetric) gene flow on deviations of the mean phenotype from the local optimum, still exists in these more complex models, but it interacts with the effect of the standing load on genetic drift, causing a sudden collapse in density at the range edge [62–64].

Models that incorporate steepening or patchy ecological gradients further showed that the simultaneous effects of gene flow on adaptive potential and on demography can be generated at very small spatial scales, by local increases in genetic variance (and therefore reductions in population density though the standing load), or by a local steepening in the gradients in the optimum phenotype [65,66]. Patchy gradients in particular may have long-term consequences on the spread of populations from their centre by local adaptation, probably due to stochasticity in cline establishment [65]. These local increases in (effective) gradient steepness can be caused by combinations of abiotic or biotic factors affecting selection, or by changes in the rates or distances of gene flow (e.g. due to the introduction of more widely dispersing pollinators or seed dispersers, changes in mating system/sexuality, or more permeable landscapes) causing alleles and genotypes to experience more environmental heterogeneity over space [8]. These models are conceptually rich in that they provide key insight into the relationships between gene flow, adaptive potential and demographic load in natural populations, and make predictions about when and where ‘swamping’ versus ‘spreading’ effects of gene flow should dominate. However, they also highlight the need to consider the life history, ecology and genomics of particular species and their communities, in order to predict when and where local adaptation to changing environments will be prevented in real situations.

This theoretical line of research has also explored how plasticity affects the interaction between population genetics and ecology. As for non-spatial models, this theory often relies on the simple assumption of linear reaction norms. When reaction norm slopes vary genetically, plasticity may evolve and thereby interact with eco-evolutionary processes in space. In particular, a quantitative genetic model assuming constant genetic variance of reaction norm slope has shown that higher plasticity can evolve in extreme environments at the limits of the range, leading to an expansion of these range limits [67]. This occurs because the variance in plasticity causes the release of cryptic genetic variance in extreme environments, therefore accelerating adaptive evolution [56,67]. This model added evolving plasticity to a model similar to Kirkpatrick & Barton’s [19] and therefore did not include the important findings about the role of drift and changes in genetic variance obtained in later theoretical studies [61–64]. However, the role of evolving plasticity in facilitating range expansion was recently confirmed in this more realistic context, using a combination of individual-based simulations and analytical results, and also allowing for (partly predictable) environmental fluctuations to further favour the evolution of plasticity in each location [68]. When the spatial gradient also moves in time (as studied without plasticity in earlier studies [26,69]), the pattern of spatio-temporal change in the environment (e.g. a similar shift in all locations vs a steepening/flattening of the gradient) determines the extent of spatial differentiation in plasticity and its contribution to niche and range limits [70].

## 3. Empirical tests of the theory

### (a) Empirical results on evolutionary rescue

Direct evidence for ER in natural populations is limited by technical challenges in demonstrating it empirically. First, because extinction is ultimately a stochastic process (the chance event of reaching zero individuals in a population), high levels of replication are required to estimate its probability. Second, even without focusing on extinction *per se*, demonstrating that observed demographic trajectories are indeed modified by genetic change due to natural selection requires tracking populations for enough time to span a substantial number of generations. Third, absence of control over the environment in the field makes it difficult to prove that any observed demographic rebound is caused by adaptive evolution, rather than a change in ecological conditions that relaxed the initial stress. Lastly, successful cases of ER may involve transient (and therefore hard to observe) population declines, where ER may only be inferred *a posteriori* as having needed to occur to maintain a population despite a previous (and demonstrably acute) stress. Such reasoning would apply to many cases of adaptation to human-imposed sources of stress, such as pesticides, antibiotics and pollution [71], but is harder to show for natural, typically multifactorial, environmental change. Regarding responses to gradual (rather than acute) environmental change, an important line of investigation in unmanipulated natural populations has been to measure the plasticity and evolvability of key adaptive traits, such as breeding time in nesting birds [72], to infer the maximum sustainable rates of environmental change. However, these studies often have to rely on somewhat strong assumptions, such as constant width and height of the fitness function across environments.

These difficulties in studying ER in natural populations probably explain why experimental work under controlled laboratory conditions is more abundant in this research area. This work has been reviewed elsewhere [52,71,73], so we will only briefly highlight the most significant or recent results here. The seminal study by Bell & Gonzalez [74] on yeast exposed to salt stress still stands out due to its large replication, made possible by the use of a liquid-handling robot, leading to robust and accurate estimates of extinction probabilities. This setting enabled the authors to demonstrate that initially larger populations were more likely to avoid extinction through ER because they more often sampled a rare rescue allele that pre-existed in the common founding population [74]. Subsequent studies on other systems investigated other limits to adaptation highlighted by theory, such as the impact of initial genetic variation [32,75,76] or the benefits of sex and recombination [77,78]. Other work has explored the genetics of adaptation in this context, namely the strength of selection on mutations that contribute to ER, compared to adaptation in non-declining populations. This question (studied theoretically by Osmond *et al.* [79]) was investigated in seed beetle adapting to a new food type, by tracking the dynamics of allelic frequency change across the genome, in order to infer the strength of selection and genetic drift at loci underlying ER [80].

The predictability of stochastic environmental fluctuations (as measured by their temporal autocorrelation) was also shown to have a strong impact on population persistence [81,82]. In a large, semi-automated experiment where the micro-algae *Dunaliella salina* was exposed to randomly fluctuating salinity with controlled autocorrelation, environmental predictability had a large influence on population growth: population growth rate increased and extinction risk decreased as autocorrelation changed from negative to highly positive [83]. This effect was mediated by (transgenerational) phenotypic plasticity, causing fitness to depend not only on the current but also on the previous salinity. Patterns of environmental fluctuations that matched the shape of a bivariate tolerance curve, with fitness as a function of past and current salinity, led to higher average population growth [83]. Plasticity and environmental tolerance also evolved in response to environmental autocorrelation in this experiment [84–86], but this evolution was not formally shown to underlie population persistence (as predicted under ER). Other experimental studies explored topics less addressed by theory, such as the influence of the demographic or evolutionary history of populations on the outcome of ER. For instance, populations of the red flour beetle *Tribolium castaneum* that experienced a bottleneck reducing their standing genetic variance were more prone to go extinct when later facing a challenging environment and had slower dynamics of adaptation when they persisted than those that were not bottlenecked [87]. Conversely, prior exposure to the same [88] or a different [89] environmental stressor was shown to increase the likelihood of ER, as did a more gradual rate of environmental change [90,91].

Most of these experimental results were obtained with microbes, with only a few studies on short-lived animals and plants (e.g. [92]). This raises the question: to what extent can conclusions drawn from experiments with microbes extend to larger and longer-lived organisms? Microbes typically have much larger population sizes than multicellular eucaryotes, rare and non-obligate sexual reproduction, smaller and simpler genomes and development and a different mode of reproduction (e.g. binary fission) and death [36,52,71]. This means that unicellular microbes are less prone to extinction and that they experience different evolutionary regimes than multicellular organisms. In fact, environmental stress generally does not lead to actual decline in many experiments with microbes but rather to severely slowed population growth, with extinction only occurring because reproduction does not compensate sufficiently rapidly for dilution upon transfer to fresh medium [74,83]. In addition, the per-generation rate of environmental change is typically slower for microbes, given their short generation times. However, an environmental change that seems slow at a coarse spatial scale (relevant to animals and plants) may also include a faster component at a finer spatial scale (relevant to microbes). For instance, higher yearly average temperature at a broad regional scale can be associated to local micro-environmental fluctuations in temperature, such that microbes may still experience rapid environmental change at their own ecological scale.

Regardless of their value in understanding the potential for adaptive responses of larger organisms, microbial responses are meaningful *per se* to understand eco-evolutionary responses to environmental change. For instance*,* phytoplankton are the primary producers of marine food webs, fungi are critical to the functioning of forests (notably through mycorrhiza), soil bacteria are essential to carbon mineralization, microbial pathogens are important regulators of populations of animals and plants, while gut microbiota are critical to digestion for many animals. Their pivotal role in many ecosystems means that although species extinction may be rarer in microbes than in larger organisms, when major changes do occur in the composition of their communities, they are likely to have large, and sometimes dramatic impacts on larger organisms in their ecosystems. The high reproductive rates of microbes, their short generation times and sometimes high mobilities also give them the potential to smooth the environment in space and time through directed dispersal, in particular up and down water columns in marine biomes in response to light and nutrient availability. At the same time, understanding the adaptive limits of long-lived eukaryotes, which typically occupy the other end of the continuum in terms of size and life history, is also highly relevant to ecosystem and climate resilience, given that long-lived and often sedentary eukaryotes are the foundation of our planet’s most biodiverse ecosystems, carbon stores and climate regulators (e.g. coral reefs, rain forests, boreal forest, etc.). This means it is crucial to understand how the environmental and demographic factors that limit ER differ across contrasting ecologies, life histories, taxa and spatial scales.

### (b) Empirical results on local adaptation at species’ range margins

Tests of adaptation at local and global ecological margins (i.e. both within species’ ranges and at their edge) were recently reviewed in another special issue of this journal [8,60,68]. One overall message is that, despite extensive research and clear theoretical predictions, powerful tests of range margin theory are difficult, largely because the estimation of key parameters is highly data intensive. Also, these key parameters vary across short timescales and distances, causing rapidly changing behaviours of populations. Conceptually, the key questions that need to be answered regarding adaptation in space and ecological expansion are: (1) how fast is the optimum changing in space relative to the environment, and how consistent is this change across generations? (2) is sufficient genetic variance available to allow trait evolution to track this changing optimum, and what is its demographic cost (standing load, figure 1)? and (3) can the growth rate of a given population at its ecological optimum allow persistence in the face of the cost of (2). As described above, an additional complication in space (relative to ER in time) is that the genetic variance in a population (2) is increased by gene flow, whenever populations differ in their mean trait values. Such gene flow may help adapt to changing environments at the range edge, as shown theoretically [69] and suggested by genomic analyses of adaptation of brown argus butterflies at their range edge [93], or may make range expansion along an existing environmental gradient more difficult, as suggested by recent experimental tests of range expansion in duckweeds [94]. Exactly how these parameters will interact depends on many values that are unknown (and difficult to know), in particular the genetic architecture of traits, their individual effects on fitness, the amount and cost of adaptive plasticity within the population, and how these parameters change in time and space.

Although genetic variance in ecological traits is almost always present in natural populations, in practice it is very difficult to measure the available genetic variation in fitness and effective steepness of ecological gradients (the basic parameters in the theory of species’ range evolution). First, how genetic variance in traits affects fitness typically differs substantially between the lab and the field [95], because more selective factors and forms of environmental variation operate in the latter. Second, genetic variances and correlations among traits (and therefore adaptive trade-offs) can vary with the environment in ways that are difficult to predict [96]. Third, genotypes rarely experience the environment passively; instead, they usually do so in a way that is directly affected by their phenotype, especially when the latter influences movement in space (by migration) or in time (by phenology) [97]. Another practical difficulty is that the actual point in space where local adaptation stops in the models described above is effectively a tipping point (where local density and genetic variation in traits or fitness become zero). This makes it difficult to predict accurately, although it may often coincide with the point in environmental space where existing plastic responses are no longer sufficient to match the local optimum (and may be actually maladaptive [98]).

It is also unclear whether the genetic variation needed for local adaptation is typically locally available (i.e. in standing variation), or instead only exists in distant populations, and therefore needs to be introduced by gene flow from other parts of the range. The dependence of local adaptation on gene flow from distant populations can be investigated by studying the distribution of structural variants (e.g. chromosomal rearrangements, §4b below) in relation to gamete/zygote dispersal (e.g. long pollen dispersal in European oaks [99]) and levels of sexuality (outcrossing in plants). Another key way forward in testing spatial theories is investigating the effect of plasticity on local adaptation, and how this changes towards range margins, where existing forms of plasticity could become maladaptive [100,101]. At the same time, however, increased genetic variance in reaction norm slopes in novel environments could cause the release of substantial genetic variance in fitness [98,102], allowing ER at range margins through the rapid evolution of plasticity [56,67].

## 4. Useful avenues for future research

In light of this synthesis of current theoretical and empirical findings on the role of adaptation limits in population persistence, we now identify key challenges and areas where future research is likely to lead to the most progress on this question.

### (a) Linking maladaptation to population dynamics

Underlying most theories on adaptation limits and extinction risk is the assumption that maladaptation, in the form of phenotypic deviations from some local optimum phenotype, has a direct detrimental effect on population growth. The rationale behind this assumption seems simple but has proven particularly challenging to demonstrate empirically, especially in a way that connects meaningfully to theoretical predictions. One reason is that, although the detrimental impacts of environmental stress on demography may be easy to observe (and even to relate to specific traits [103,104]), they cannot always be ascribed to maladaptation that may be overcome by genetic change over micro-evolutionary timescales. Some detrimental demographic effects of the environment instead result from so-called ‘hard limits’ [8] (also described as ‘habitat quality’ effects [101,105]; figure 1), which would require fundamental changes to physiology (or phenotypic or social organization) that typically take place over macro-evolutionary timescales (e.g. key innovations or critical transitions). In addition, the current state of a population (in terms of allele frequencies, traits and fitness) always reflects past as well as ongoing selection and is also often influenced by gene flow from other populations. This means that approaches for inferring phenotypic selection [106,107], even when explicitly accounting for temporal changes in the fitness function [108], do not necessarily inform us about the causes of maladaptation at any given time.

Another difficulty with estimating the demographic consequences of population genetics is that the real demography that underlies fitness in natural populations includes complexity that is missing from most evolutionary demographic models. In most of the theoretical literature summarized above, all of the influence of maladaptation on population dynamics is summarized by its effects on a phenotype-dependent intrinsic rate of increase (sometimes combined with phenotype-independent density regulation, e.g. [19,37]), with little attention paid to where in the life cycle maladaptation has most impacts on demography. This approach overlooks the possibility for demographic compensation to take place among different life-history components of fitness. For example, in a Dutch population of great tits, mismatches between the realized and the optimal laying date led to substantially reduced fertility, but with no net effect on population growth (and in particular, no decline). This result was all the more striking given it concerned reproductive phenology, the category of traits for which most plastic and evolutionary responses to climate change are documented [109,110] and with clear evidence for a moving optimum phenotype as assumed in many theoretical models [108,111]. The reason for this discrepancy was that the surviving offspring from one breeding season experienced relaxed competition and thus had a higher recruitment rate in the next breeding season [112,113]. The influence of maladaptation on population growth was therefore buffered by demographic compensation among different life-history components of fitness. What made this outcome possible was the combination of: (i) excess fertility, where substantially more offspring are produced than are needed for replacement; (ii) an episode of selection limited to a specific component of the life cycle (breeding season); and (iii) (st)age-specific density-dependent competition. Disrupting any of these elements could increase the net demographic impact of maladaptation. For instance, if environmental degradation on other niche axes (not directly related to breeding time) caused the number of offspring to barely allow replacement when the mean laying date is optimal (i.e. small excess fertility), then a maladapted laying date would be less likely to be fully compensated by higher recruitment. This example illustrates how the intricacies of the life cycle, and the way selection is incorporated therein, determine the influence of adaptation limits on extinction risk in a changing environment.

Beyond these difficulties in assessing the demographic consequences of maladaptation, evaluating maladaptation itself and how it depends on the environment also may be difficult. This is especially true when the direction of selection on a particular trait (or set of traits) conflicts among life-history stages [114]. Evolution of traits involved in such selective conflicts underlies the evolution of trade-offs [16] and may lead to evolutionary life-history shifts in a changing environment (e.g. increased fecundity at the expense of adult survival) that challenge interpretations in terms of adaptive strategies [115], especially when not all relevant life-history components of fitness are analysed. The strength and direction of selection may also change with population density [116,117], which could even lead to situations where population decline is causing maladaptation, rather than the reverse [118]. Such reciprocal causation is the hallmark of any eco-evolutionary feedback [119], for which dedicated statistical approaches [120], ideally combined with manipulative experiments, are required to establish significant causal paths and their magnitudes, for example, by showing that evolutionary change in a trait causes ecological change, which then modifies selection on the same trait.

### (b) Genetic and genomic architecture of adaptation

Understanding what limits the rate of adaptation also requires understanding how genetic variation in fitness is determined. That the rate of change in fitness by natural selection equals the additive genetic variance in fitness was formalized about a century ago by Fisher’s fundamental theorem [121] and later extended to phenotypes by Robertson and Price [122,123]. Since then, a large body of research has been dedicated to understanding how the dynamics of adaptation is constrained by its genetic basis. Although we cannot cover this topic extensively here, we briefly highlight the key elements that most affect our ability to predict adaptation and population persistence in new environments.

Many types of environmental changes (such as rising temperature) exert natural selection on polygenic traits determined by a large number of loci and alleles spread throughout the genome. When these traits are identified, their evolution can be studied using quantitative genetics, estimating the heritable component of phenotypic variation while ignoring its genetic details [124,125]. However, under strong natural selection (as expected under drastic environmental change), large changes in allelic frequencies at individual loci and associations among loci (linkage disequilibrium) can cause rapid changes in the genetic variances, correlations and distributions of traits [126,127], compromising simple quantitative genetic predictions that assume a constant genetic covariance matrix **G** [128]. The extent to which genetic (co)variances will change over time crucially depends on how many loci contribute to the trait affecting fitness in a given generation, and with what distributions of phenotypic effects. All else being equal, traits determined by fewer loci, and with more heterogeneous distributions of phenotypic effects among loci (i.e. with less even genetic architecture), should undergo more rapid evolutionary changes in genetic variances and covariances in a novel environment, although this has not been studied in much detail. Another aspect of genetic architecture that is likely to matter considerably is the relationship between the initial frequency of beneficial alleles and the size of their phenotypic effects. The same amount of genetic variance could be explained by low-frequency alleles of large effect, or mid-frequency alleles of smaller effects, but the implications are not the same for the response to selection [129]. This joint distribution of allelic frequencies and phenotypic effects is the result of the evolutionary history of the population. If the environment has been stable for a long time prior to changing, then most alleles that might rescue the population following the onset of stress are likely to have been deleterious before and maintained at mutation-selection(-drift) equilibrium, with a frequency inversely proportional to the size of their fitness effect [32,33,126,130]. These details of genetic architecture will influence how fast a population can adapt to a changing environment [130,131].

The genomic architecture of adaptation is also receiving increasing attention, given its influence on the efficiency of selection, and therefore on the rate of adaptation. Structural variants, such as inversions or other types of chromosomal rearrangement (fusions, translocations and duplications), are documented in a growing number of case studies of adaptive evolution, where they are thought to be favoured because the local reductions in recombination rates they provoke create supergenes that tie together alleles at different loci that are beneficial and work well together [132–134] (although selection specifically at inversion breakpoints could also play a role [135]). Reductions in recombination also decrease the genetic variance of traits that are selected for an optimum phenotype (as in figure 1), by favouring the maintenance of negative associations between loci generated by stabilizing selection (Bulmer effect [136]). This reduction of genetic variance of traits may be favoured if it reduces the standing load, as suggested by the apparent maintenance of chromosomal rearrangements and structural variants in local adaptation [137]. If such structural variants arise and spread while a population is adapting to a new or changing environment, they can dramatically modify the genetic architecture of ongoing adaptation, by introducing a gene with a major effect on genetic variances and correlations among traits. This could cause a rapid reorientation of evolution [137], with potentially unpredictable outcomes such as evolution towards a different fitness peak [138]. A crucial question then is: how rare or unlikely is the creation of such structural variants by mutation and/or their maintenance as standing variation in populations, allowing their rapid spread during adaptation to a new environment? In other words, how rapidly can genomic architecture itself evolve in response to the demands of environmental change, changing future evolution trajectories?

Another critical question regards the genetic basis of genotype-by-environment interactions, which underlie genetic variance in plasticity [139–141]. Many quantitative genetic models of plasticity simply assume that genetic variances and covariances exist for reaction norm parameters such as their slopes (a metric of plasticity in linear reaction norms [142]) or for trait values in different environments (so-called ‘character states’ [139,143]). How (and how quickly) these genetic (co)variances themselves evolve, and how this impacts responses to selection in variable environments, remains essentially an empirical question (e.g. [96,144]), which depends on the underlying genetic basis of plasticity [141,145]. In particular, plasticity in gene expression underlies the plasticity of many higher traits, and a growing number of studies have investigated how such gene expression plasticity varies genetically, and the extent to which it predicts the direction of evolution in gene expression (reviewed in, e.g. [146,147]). A major challenge is now to relate variation in plasticity for gene expression to that for traits that are directly connected to fitness and population growth [96,98]. A key step in that direction will involve characterizing the stability and evolvability of gene-regulatory networks across environments and genotypes [146,148].

### (c) The dynamics of (transgenerational) plasticity

Another emerging research line on plasticity concerns its dynamics. Most models of evolutionary demography that incorporate the evolution of plasticity assume that the environment influences the phenotype once during development and that this phenotype also influences fitness and population growth only once [3,37,49,50]. In other words, they ignore the possibility for plasticity itself to be dynamic, both within and between generations, and for natural selection on the expressed plastic trait to also change over the life cycle in response to the environment. It is however clear that many plastic traits change within generations through continual interactions with the environment, with rates and patterns (lags, maximum response, etc.) that can be quantified empirically [149–151] and that may vary genetically, and therefore evolve. Over longer time scales, transgenerational effects of environments on traits can also accumulate [152] or interact [153] among generations, making them difficult to distinguish from the cumulative effect of evolutionary change. Accounting more explicitly for the dynamics of plasticity (as advocated recently [149–151]), including by analysing how transgenerational effects unfold along a lifetime [154,155], would improve our ability to predict eco-evolutionary responses to changing environments.

Theory has begun to investigate the evolution of plasticity for labile, reversible traits [156–158] and to characterize how selection acts on age-dependent plasticity [159]. However, we still lack theoretical predictions for how such dynamic (i.e. continually revised) plasticity and its evolution influence population growth and extinction risk in a changing environment. In particular, environmental predictability (e.g. autocorrelation), an important driver of eco-evolutionary dynamics (as emphasized above), has a stronger influence on the evolution of plasticity for a fixed than for labile plastic trait [56,156]. This is because a labile trait is likely to influence fitness at multiple points in time, meaning that mismatches between current cues and future selective conditions become less crucial [156]. On the other hand, a trait that is fixed early in development may allow a greater change in trait magnitude (i.e. a steeper reaction norm) in response to the environment compared to a trait that has to remain labile for a sustained period of sensitivity throughout a genotype’s lifetime [97]. The impact of environmental predictability on phenotypic lags and mismatches, and their cascading effects on population dynamics through maladaptation, will therefore differ between labile and fixed plastic traits, but this has been little investigated theoretically.

These dynamic aspects of plasticity may also tilt the balance between the benefits and costs of plasticity. Costs of plasticity have been postulated theoretically [160] and formalized conceptually [161], but empirical measurements suggest they are overall small [162]. However, these costs may become more substantial for reversible plasticity, including the costs of extending windows of sensitivity, potentially reducing the breadth of phenotypic responses [97], or the metabolic costs of repeatedly changing the phenotype. To our knowledge, these costs of reversibility have rarely been measured, but they are suggested by the fact the most flexible forms of plasticity (such as cognition) are typically restricted to taxa facing situations and environments that are highly dynamic, predictable only over short timescales, and that demand continual revision of a given phenotype through its sensitivity to the environment. Ultimately, the evolution of dynamic phenotypic plasticity is likely to be driven by a trade-off between the limits of irreversibility—producing fixed phenotypes that may be unfit under future environmental conditions—and the cost of phenotypic reversibility [97], but this process has been little investigated theoretically (but see [156–158]).

### (d) Adaptation to the abiotic environment versus to other species

Our synthesis so far has focused on populations of individual species, rather than communities or ecosystems. However, even when considering how a focal species responds to a change in its abiotic environment, many of the responses we discuss will be driven by interactions with other species that are affected by this environment, rather than the abiotic environment itself. For many species, the main limiting factor and selective pressure under climate change (and other types of environmental change) is likely to involve other species, from scarcity of resources to changes in competition [163], predation [164], mutualism [92] or herbivory [93,165].

Conceptually, interacting species should be considered as part of the environment, meaning that the biotic environment is also covered by theory on adaptation to a changing environment (e.g. based on a moving optimum). However, adaptation to interacting species is more prone to eco-evolutionary feedbacks and transient dynamics [119], where the evolution of a focal species can modify the selective pressure that it itself experiences. This can undermine our ability to test predictions for evolutionary responses to environmental change within species. For instance, even in simple food webs with only two prey and two predators, ER is most likely to be indirect and mediated by the evolution of other species [166]. Ecological interactions are also likely to modify the shape of fitness landscapes. For instance, trait-mediated resource competition can cause disruptive selection, favouring extreme rather than intermediate phenotypes [167]. Even when the overall shape of the fitness landscape is unchanged, a competitor species can lead the mean phenotype of a focal species to deviate from an optimum determined by the abiotic environment alone, and such character displacement can in turn affect gene flow, local adaptation and evolution of range limits over an environmental gradient [168]. This process is further modulated by phenotypic plasticity and its effects on environmental tolerance (and thereby on local population density), which is likely to have cascading effects on species richness and overlap over the environment gradient [169].

The ultimate challenge is to understand how the adaptive limits of multiple species, in response to both the changing environment and their mutual interactions, determine changes in community compositions and ecosystem functioning under environmental change. While a detailed understanding of such dynamics from first principles is probably beyond reach, both empirical [170] and conceptual [30,171] work has started predicting the determinants of ER across entire communities, generating hypotheses that can be refined, extended and tested in future research.

## 5. Conclusions

### (a) Focusing empirical studies on common species in ecosystems

The empirical approaches we summarize above are time-consuming and costly, and demand extensive basic and labour-intensive research. Such a detailed understanding clearly cannot be reached for most species with conservation concerns. In addition, most species on the brink of extinction (i) are too rare to be studied in sufficient numbers to obtain the demographic, genetic and ecological parameters needed to predict ER; and (ii) usually have more pressing issues that need to be addressed before focusing on evolution, such as habitat restoration and connectivity. This highlights a key lesson from Georgina Mace’s career and her practice of conservation biology: a commitment to understanding the fundamental processes that drive the evolution and ecology of species in communities, rather than only maximising the protection of charismatic species that have recently become rare.

In order to understand these processes, we need to test how the common species that largely drive community resilience persist throughout their geographical range and how this persistence depends on adaptive evolution (i.e. population genetics), as well as plasticity. Laboratory and field experiments, together with genomic analyses of naturally abundant and naturally rare species across life histories and taxa (rather than species that have recently declined in abundance) will help understand when and where the additional effort involved in combining evolutionary ecology and genetics with demography is most useful. In particular, this approach can identify which rates of environmental change in space or time lie beyond what we can expect unassisted ER to accommodate—in terms of maintaining ecosystem outputs or persistence of key species—and help predict tipping points for biodiversity. Such an approach has real economic and societal importance (e.g. for food security, water supply and infrastructure resilience) and needs to be central to conservation biology, alongside focusing on rare species that have become of conservation interest for other reasons.

### (b) Predicting adaptive potential in the novel environments of coming decades

The value of the theory and empirical tests for ER that we have summarized lies in the insights they provide regarding: (i) the relative contributions of standing versus *de novo* genetic variation; (ii) how the genomics of traits affecting fitness determines adaptation to different forms of environmental change; (iii) the temporal and spatial scale at which environmental change shapes fitness variation within population; and (iv) how phenotypic plasticity and its evolution modify these processes. A key lesson from such insights is to renew focus on variation within (rather than mostly across) species, and its importance in allowing niche evolution in response to environmental change. This is especially important for ecosystems that are key to ecological resilience but that are only now open to study through modern genomic techniques (e.g. metagenomics and eDNA) and high-throughput biodiversity monitoring through, e.g. artificial intelligence analyses. What is clear is that evolutionary responses (or lack thereof) will become increasingly crucial in predicting ecological resilience, given virtually all populations and ecosystems will soon be exposed to environments that lie significantly beyond those they have evolved to cope with, even across their geographical range.

## Data Availability

This article has no additional data.
